# Childhood Trauma and Aggression in Persons Convicted for Homicide: An Exploratory Study Examines the Role of Plasma Oxytocin

**DOI:** 10.3389/fpsyt.2021.719282

**Published:** 2021-08-17

**Authors:** Kah Kheng Goh, Mong-Liang Lu, Susyan Jou

**Affiliations:** ^1^Department of Psychiatry, Wan-Fang Hospital, Taipei Medical University, Taipei, Taiwan; ^2^Psychiatric Research Center, Wan-Fang Hospital, Taipei Medical University, Taipei, Taiwan; ^3^Department of Psychiatry, School of Medicine, College of Medicine, Taipei Medical University, Taipei, Taiwan; ^4^Graduate School of Criminology, National Taipei University, Taipei, Taiwan

**Keywords:** oxytocin, childhood trauma and adversity, homicide, aggression, violence

## Abstract

Evidence has demonstrated the association between childhood trauma and criminality in adulthood, however, less is known about how best to explain the route from childhood trauma to adulthood aggression. Results from both human and animal studies have generated the hypothesis that dysfunction of the oxytocinergic system may correlate with pathological aggression. The current study represents a first exploratory examination to investigate the trajectory from childhood trauma to aggression, specifically, plasma oxytocin's role in this association. We assessed the childhood trauma experiences in a total of 108 participants, including 33 persons convicted for homicide and 75 non-offending healthy participants, using the Childhood Trauma Questionnaire, with in-depth clarification interviews for cross-validation. All participants were checked for aggression using the Modified Overt Aggression Scale and their plasma oxytocin levels were obtained. Results indicated that persons convicted for homicide had higher childhood trauma scores and lower plasma oxytocin levels than healthy controls. The plasma oxytocin levels were inversely correlated with childhood trauma in all participants. Further mediation models were constructed to explore these associations, in the best-fit model, the relationship between childhood trauma and aggression is mediated by plasma oxytocin levels in persons convicted for homicide. In conclusion, the association between childhood trauma and aggression of persons convicted for homicide is mediated by their plasma oxytocin levels. With leading to further theoretical consideration in the causality on how best to explain the interaction between childhood trauma and aggression, the current study may assist in developing further research and preventive strategies for aggression, particularly the importance of early identification of childhood trauma.

## Introduction

Violence remains a leading cause of mortality worldwide. The burden and harm elicited by violent crimes are tremendous, engendering a combined negative effect on society in terms of both insecurity and physical disability. Considering the relation to offender characteristics and violent crime formation models, recent studies have reported that most offenders have a history of childhood trauma (1, 2). Trauma experienced in childhood has severe consequences. Individuals with childhood trauma are at higher risk of engaging in problematic behaviors; subsequently resulting in adverse effects, on both physical and mental health, that may appear in childhood and continue into adulthood (3, 4). Although several studies using forensic and psychiatric samples have suggested a relationship between childhood trauma and violent crime (5–7), less is known about how best to explain the route from childhood trauma to adulthood aggression. Various brain circuits, including the amygdala and pre-frontal cortex, may be involved in aggression formulation (8). In addition to these anatomical findings, neurotransmitter activity, such as serotonin, dopamine, norepinephrine, and γ-aminobutyric acid, is proposed to be positively correlated with aggression (8). Childhood trauma compromises homeostasis and leads to numerous psycho-neuroendocrine changes that may affect physiological, emotional, cognitive, and social functioning, including the ability to regulate, affect, and subsequently develop empathy (9, 10).

Oxytocin plays a crucial role in stress and aggression, with animal studies demonstrating its association to maternal behavior, aggression, non-social behaviors (11), and stress response regulation (12). Oxytocin lowers hypothalamus–pituitary–adrenal activity and cortisol levels, which increases levels of plasma oxytocin; this results in a negative feedback system, where stress increases the level of cortisol, which in turn increases the level of oxytocin, resulting in a subsequent decrease in cortisol (13). History of aggression was shown to inversely correlate with oxytocin levels in the cerebral spinal fluid, indicating that oxytocin plays a mechanistic role in human aggression (14). In one study, the fight-or-flight response increased activation of the amygdala in participants with low oxytocin levels, which was associated with a lack of trust (15) in children who had experienced trauma. The association of oxytocin with trustworthiness was also demonstrated in an experimental adult's monetary payoffs study (16). Low plasma oxytocin levels have been observed in children who experienced trauma (17) and in adults who were exposed to childhood trauma (18). Further, disrupted oxytocin regulation was noted in individuals with childhood trauma (19). The severity of childhood trauma may also have an inverse correlation role in modulating oxytocin concentrations (20). It is possible that individuals who have experienced trauma do not have a normally functioning oxytocin inhibition process, allowing stress responses to escalate to unproportionally high levels (13). This phenomenon may also be caused by a failure to exhibit high oxytocin receptor levels in the amygdala of children with experiences of trauma. A low oxytocin level may be associated with neurostructural changes in those who experienced maltreatment, for example, a low oxytocin level is associated with larger hypothalamus and amygdala volumes (21) as a result of the compensatory growth mechanism. Reduced amygdala activation, under the influence of oxytocin, has been shown to reduce danger signaling, and is linked to the promotion of trust, increased sociability, and decreased social fear (22). Lower salivary oxytocin levels in maltreated children lead to a lower percentage of gaze fixation for the human face eye area and this visual attention deficit is resulting in social-emotional problems (23). It is believed that untoward childhood trauma may interfere with the oxytocinergic system on a more fundamental level, particularly affecting the molecular and genetic mechanisms. Genetic variation, of the oxytocin receptor (Oxtr) gene for example, may moderate the link between childhood trauma and social relationship in adulthood (24). The total Oxtr-knockout male mice (excised at the time of conception) had heightened aggression compared with the controls while the predominantly forebrain-specific Oxtr-knockout male mice (excised postnatally) displayed similar aggression levels with controls mice (25). This animal study indicates that oxytocin may play an important role in the development of neural circuits that underlie aggression in adulthood. In the human study, the single-nucleotide polymorphisms of the Oxtr gene, for example, rs7632287 (26) and rs53576 (27) were associated with the frequency of aggressive behaviors.

Supplementation of exogenous oxytocin has been shown to attenuate the amygdala's response to social stress and fear (28). Exogenous oxytocin induces a momentary “state of mind” change in individuals through a reduction in feelings of fear (29) and resulting in an alteration to the brain's cognitive–emotional schemas and shifting an individual's perception of others as untrustworthy to more trustworthy (30). Intranasal oxytocin administration decreased social threat hypersensitivity and, accordingly, reduced both anger and aggressive human behaviors (31). In the study of task-related aggressive responses, oxytocin administration decreased aggression in healthy young men and the higher baseline endogenous urinary oxytocin levels were associated with less aggressive responses (32). In a study of general healthy participants, compared with a placebo group, behavioral aggression was slightly higher in the experimental group directly after the intranasal administration of oxytocin, although the opposite was found as the study period progressed (33). In another study of healthy participants, compared with the placebo group, the intranasal administration of oxytocin increased the aggressive responses of participants in the experimental group while playing a monetary game (34). The acute effect of oxytocin on aggressive behavior did not been observed in another study of the healthy adult man but when examining those responders, higher scores on antisocial personality traits were related to their increase in aggression response following oxytocin administration (35).

All of the evidence implies that endogenous and exogenous oxytocin may be capable of modulating aggressive behaviors in humans. However, the link between downregulation of the oxytocinergic system and heightened aggression is less straightforward and required further research (36). Various experimental studies try to postulate the pathways and possible factors that may tangle between childhood trauma, aggression, and oxytocinergic dysfunction. Despite this evidence, results of research investigating the correlation between childhood trauma, oxytocin levels and aggression are, in general, mixed and inconsistent.

The role of oxytocin in persons convicted for homicide is the primary focus of our study. Childhood trauma may disrupt oxytocin regulation, with a decrease in oxytocin levels potentially correlating with heightened levels of aggression. However, if oxytocin mediates the route from childhood trauma to adulthood aggression is still unknown. This study aims to explore a theoretical framework that may explain the pathway through which childhood trauma leads to aggression in persons convicted for homicide. First, it is hypothesized that persons convicted for homicide have higher scores in childhood trauma measures compared with those healthy participants. Further, we postulate that persons convicted for homicide have lower plasma oxytocin levels than healthy participants who have not committed violent crimes. In addition, under the presumption that aggression is a phenotype associated with persons convicted for homicide and manifests in them committing violent crimes, this study aimed to examine the proposed trajectory from childhood trauma to adulthood aggression, oxytocin is considered to contribute to aggression.

## Methods

### Participants

This study was conducted between November 1, 2018, and April 30, 2019, following approval from Taiwan's Ministry of Justice. Persons convicted for homicide (Taiwan Criminal Codes §271, §272, §273, and §274) were recruited from probation offices in Taipei, Shihlin, Taoyuan, and Hsinchu, while control group participants were recruited from the community through research advertisements, and were without a history of criminal convictions, illicit drug use, and mental disorders. A total of 108 participants, composed of 33 in the homicide group and 75 in the control group, were enrolled. The demographic data of participants and their related characteristics are shown in Table 1. All participants were aged 20–65 years, male, had adequate mental competence, and were willing to provide written informed consent. Those who had received hormone therapy were excluded. All participants were interviewed by qualified psychiatrists using structured interviews for the screening and diagnosis of mental disorders. To minimize the possible confounding effects of current trauma-related symptom severity and other psychopathology, none of the participants had schizophrenia, bipolar disorder, posttraumatic stress disorder, epilepsy, intellectual disability, dementia, neurocognitive disorder, or other serious medical illnesses. Explanations regarding the purpose, content, process, and possible risks involved in this study were delivered orally to all participants. Participant rights were thoroughly explained, particularly, that their criminal sentences, parole or probation periods would not be affected by whether they chose to participate in the study or not, and written informed consents were collected. Participants were free to withdraw from the study at any time. Recruited offenders continued to serve their sentences, with their probation periods unchanged. This study was approved by the Joint Institutional Review Board of Taipei Medical University.

**Table 1:** Demographic characteristics of all participants.

	**Homicide group (** ***N*** **= 33)**		**Healthy group (** ***N*** **= 75)**		***p***
	***M***	***SD***	***M***	***SD***	
Age (in year)	39.97	12.44	39.05	12.48	0.73
Height (in cm)	170.18	7.15	173.15	5.75	0.02
Weight (in kg)	72.82	19.97	69.16	11.04	0.23
Education (*n*, %)					<0.001
University	4 (12.1)		41 (54.6)		
High School	16 (48.5)		27 (36.0)		
Middle School	10 (30.3)		5 (6.7)		
Primary School	3 (9.1)		2 (2.7)		
Marital Status (*n*, %)					<0.01
Single	22 (66.6)		48 (64.0)		
Cohabit	4 (12.1)		2 (2.7)		
Married	2 (6.1)		22 (29.3)		
Divorced	5 (15.2)		3 (4.0)		
Age at index offense (in year)	29.82	11.26			
Length of sentences (in year)	12.85	7.95			
Length of imprisonment (in year)	8.46	5.76			
Alcohol drinking (*n*, %)					<0.001
No drinking	11 (33.3)		50 (66.7)		
Low risk drinking	5 (15.2)		23 (30.6)		
Risky alcohol drinking (≥14 drinks/week)	17 (51.5)		2 (2.7)		
Overall self-rated health condition					0.04
Very poor	3 (9.1)				
Poor	3 (9.1)		2 (2.7)		
Equivocal	8 (24.2)		27 (36.0)		
Good	16 (48.5)		39 (52.0)		
Very good	3 (9.1)		7 (9.3)		
Cigarette smoking (*n*, %)	26 (78.8)		6 (8.0)		<0.001
Cigarette consumption (in pack per day)	0.64	0.52	0.02	0.09	<0.001

### Measures

#### Demographic Questionnaire

The first part of the questionnaire related to participants' basic demographic data, including height, weight, age, date of birth, place of birth, marital status, educational history, occupational history, family history, medical history, psychiatric history, current medication use, alcohol and cigarette use, and self-reported criminal convictions. Additional information on age at index offense, length of sentence, and length of imprisonment were collected through questionnaires provided to persons convicted for homicide only.

#### Childhood Trauma

Childhood trauma is defined as child maltreatment constitutes all forms of ill-treatment, abuse, neglect or negligent treatment or commercial or other exploitation, resulting in actual or potential harm to the child's health, survival, development or dignity in the context of a relationship of responsibility, trust or power (37). The Childhood Trauma Questionnaire-Short Form (CTQ-SF) (38) was used to assess the childhood trauma of participants. In a previous study, the CTQ-SF was translated into Chinese, and the translated version's reliability was confirmed (Cronbach's α = 0.57–0.90; Intra-class coefficient = 0.67–0.85) (39). The CTQ-SF screens the history of participants for childhood adversities and consists of 28 items, measuring five types of aforementioned childhood trauma. Participants with scores exceeding the moderate exposure cutoff point on each subscale (physical abuse: ≥10; emotional abuse: ≥13; sexual abuse: ≥8; physical neglect: ≥10; emotional neglect: ≥15) were classified as having a history of exposure to childhood trauma (19, 40). Physical, emotional, and sexual abuse were further categorized as major childhood trauma. For cross-validation, participants were asked to provide detailed clarification about their childhood trauma experiences during individual interviews.

#### Aggression

Aggression is defined as an intention to harm another person who is motivated to avoid that harm and the perpetrator has strong faith that the behavior will harm the target (41). Besides the homicide crimes convicted by the offenders, the characteristics of aggression of all participants were also been measured using both the Modified Overt Aggression Scale (MOAS) (42), a 4-part behavior rating scale designed to measure four types of aggressive behaviors; namely, verbal aggression, aggression against property, auto-aggression, and physical aggression. Participants were asked to determine whether each statement appropriately described their behaviors over the past week, and during the most serious incidents of their lifetime. The reliability and validity of the Chinese versions of MOAS were assessed in previous studies (Intra-class coefficient = 0.94; Mann-Whitney test *Z* = −2.89) (43).

#### Oxytocin Laboratory Assessment

Ten milliliters of venous blood were collected from each participant's antecubital region or hand. Plasma oxytocin levels were determined using an enzyme immunosorbent assay (Catalog number: EKE-051-01, Phoenix Pharmaceuticals, Inc., Burlingame, California, USA) with an oxytocin detection range of 0–100 ng/mL. Each plasma sample was assayed twice, and the mean of the two measurements used for analysis; with intra- and inter-assay coefficients of variation both being <5%. No significant cross-reactivity or interference between oxytocin and analogs was observed.

### Statistical Analysis

All collected data were transcribed to SPSS Statistics version 25.0 (IBM Corporation New York, USA), for coding and analysis. Data ranges for each variable were checked to ensure they adhered to the prescribed range for each questionnaire manual. Descriptive statistics were applied to summarize the demographic characteristics and psychometric measurements of participants. Continuous variables were expressed as means with standard deviations, whereas categorical data were presented in numbers and percentages. The Kolmogorov–Smirnov test was performed to determine the normal distribution of participant age. Levene's test was used to determine the homogeneity of variances. For descriptive statistics and outcome measurements, an independent sample *t*-test was used to evaluate continuous variables; whereas Pearson's chi-square test was used to evaluate categorical variables and compare demographic variables among two groups. To investigate our theory-based hypotheses, a bivariate Pearson correlation analysis was used to estimate correlations between outcome measurements. Outcome measurements were included in a mediation analysis if all their correlations were statistically significant. Age, height, body weight, and cigarette smoking, which may interact with plasma oxytocin levels, were included as covariates in the mediation analysis model. For mediation analysis, SPSS macro PROCESS v3.3 (model 4) was applied to analyze three significant outcome measurements. Mediation effects were reconfirmed using a structural equation model, which was analyzed using the SPSS Amos 26.0 software program. Regression (path) coefficients were all in unstandardized form, as standardized coefficients generally have no use in substantive interpretations (44). A positive regression coefficient implies that a unit increase in a variable leads to a direct increase in the variable it is projected to, proportional to the size of the coefficient; vice versa for a negative coefficient. Thus, the extent of change in a dependent variable (aggression), when one unit of the independent variable (childhood trauma) increases under the condition of an unaltered mediator variable (oxytocin), is considered to be due to a direct effect in mediation analysis. An indirect effect is the extent to which a dependent variable changes when the independent variable is held constant, and changes in the mediator variable are consistent with increases of one unit in the independent variable. In other words, an indirect effect is the extent of mediation. The total effect is the sum, or modified combination, of direct and indirect effects in this study. Bootstrapping, which is considered the most effective method to use with small samples and has the lowest susceptibility to type 1 error, was used to assess the mediation effect's stability (45). Both bias-corrected and percentile-method bootstrapping were conducted, with the data resampled 5,000 times. Statistical significance for all tests was represented by a *p*-value < 0.05.

#### Models

As all measures of childhood trauma and aggression were collected retrospectively, in terms of causal relationships, we examined these variables in different models, to illustrate possible links between variables. In Model 1, as proposed according to our theoretical hypothesis, we examined the mediating role of oxytocin between childhood trauma and aggression. In Model 2, childhood trauma and oxytocin both contributed to aggression independently. In Model 3, childhood trauma was linked to both oxytocin and aggression independently. In Model 4, both childhood trauma and aggression contributed to changes in oxytocin, with childhood trauma also leading to aggression. In Model 5, childhood trauma was linked to aggression, with aggression subsequently promoting changes in oxytocin. In all tested models, model fit was examined using a goodness of fit index (GFI) ≥0.90, and a root mean square error approximation (RMSEA) value of ≤ 0.06 (46). The best-fit model was selected by considering all criteria in this study.

## Results

### Demographic Characteristics

The proportion of graduates from higher education institutions was significantly lower in the homicide group than in the control (*p* < 0.001). The prevalence of alcohol drinking and cigarette smoking was higher in the homicide group than in the control group (*p* < 0.001). Notably, almost half of the participants in the homicide group had risky alcohol drinking problems. A higher proportion of participants in the homicide group rated their health condition as poor or very poor (*p* < 0.05) (see Table 1).

### Childhood Trauma

The total scores of CTQ-SF among persons convicted for homicide were higher than those of participants in the control group (*p* < 0.001). Participants in the homicide group surpassed participants in the control group in terms of the prevalence of and the proportion screened positive for physical abuse, emotional abuse, physical neglect, and emotional neglect, except for sexual abuse. Both the prevalence of major childhood trauma (*p* < 0.01), and the mean number of trauma types (*p* < 0.001), were higher in the homicide group than control (see Table 2).

**Table 2:** Childhood trauma and measures of aggression in all participants.

		**Homicide group (** ***N*** **= 33)**		**Control group (** ***N*** **= 75)**		***p***
		***M***	***SD***	***M***	***SD***	
***Childhood trauma***						
Positive for childhood trauma (*n*, %)	Physical abuse	13 (39.4)		6 (8.0)		<0.001
	Emotional abuse	6 (18.2)		2 (2.7)		<0.01
	Sexual abuse	5 (15.2)		7 (9.3)		0.38
	Physical neglect	32 (97.0)		54 (72.0)		<0.01
	Emotional neglect	13 (39.4)		9 (12.0)		<0.01
	≥1 type of major trauma	16 (48.5)		11 (14.7)		<0.01
CTQ-SF	Total scores	58.85	14.67	46.87	9.89	<0.001
	Physical abuse	9.15	4.54	6.52	2.18	<0.001
	Emotional abuse	8.79	3.81	6.92	2.19	<0.01
	Sexual abuse	6.12	2.43	5.48	1.13	0.06
	Physical neglect	13.91	2.71	11.16	2.06	<0.001
	Emotional neglect	12.15	4.50	9.37	3.06	<0.01
Number of childhood trauma types		2.09	1.71	1.04	0.88	<0.001
***Aggression***						
MOAS	Total scores	28.61	7.63	8.75	8.85	<0.001
	Physical aggression	4.00	0.00	1.03	1.24	<0.001
	Verbal aggression	3.00	1.12	1.15	1.20	<0.001
	Aggression against object	2.39	1.50	0.91	1.15	<0.001
	Auto-aggression	1.39	1.68	0.56	0.99	0.02

### Aggression

Participants in the homicide group had a higher total score for the history of incidents of aggression (measured by the MOAS; *p* < 0.001) and had more incidents of all four types of aggressive behaviors, than those in the control group (see Table 2).

### Plasma Oxytocin Levels

**Figure 1:**
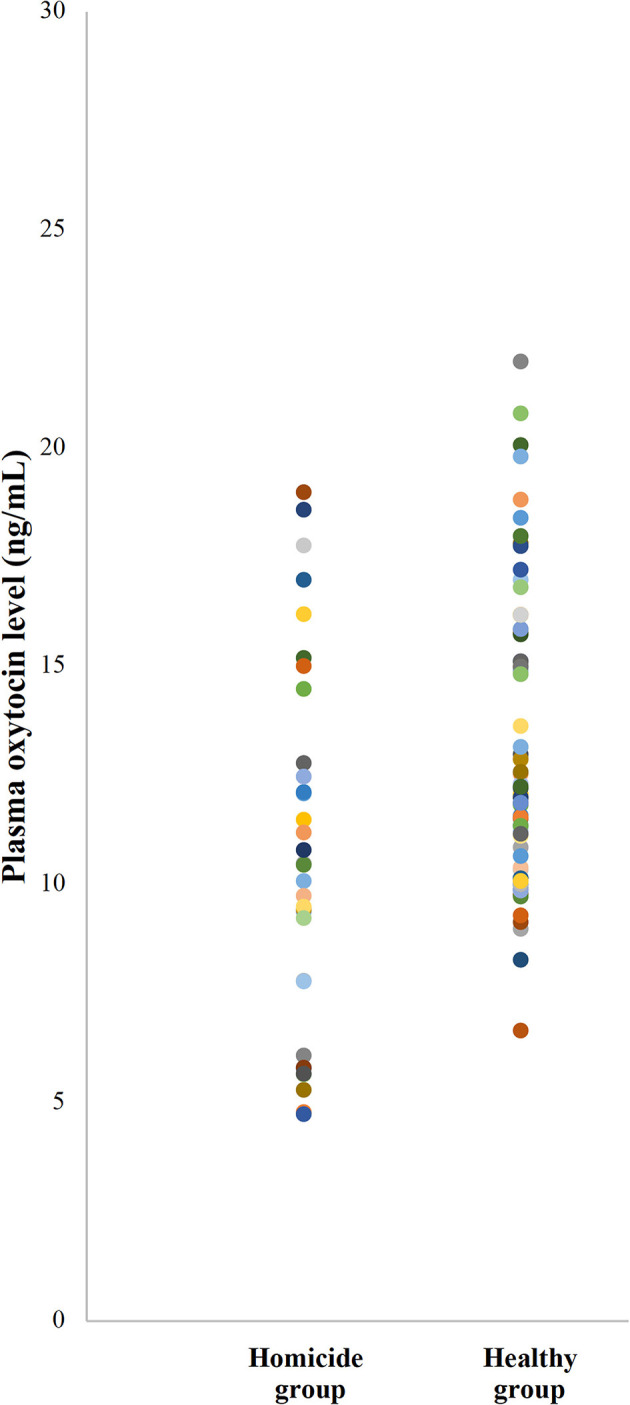
Scatter plot illustrating the plasma oxytocin levels of participants in homicide groups and healthy groups. Each color dot represents the plasma level of oxytocin of each participant.

The plasma oxytocin levels in participants of different groups are illustrated in Figure 1. Overall, plasma oxytocin levels were not correlated with age (*r*(107) = −0.15, *p* = 0.60). Participants in the homicide group (*M* = 10.74, *SD* = 4.19, 95% CIs [9.25, 12.22]) had lower levels of plasma oxytocin than those in the control group (*M* = 15.49, *SD* = 6.00, 95% CIs [14.11, 16.87]) (*p* < 0.001).

### Interaction Between Variables in a Zero-Order Correlation

Childhood trauma was significantly associated with aggression, both in the homicide group [*r*(32) = 0.48, *p* < 0.01] and the control group [*r*(74) = 0.49, *p* < 0.001]. Plasma oxytocin levels were inversely correlated with childhood trauma in both the homicide group [*r*(32) = −0.45, *p* < 0.01], and the control group [*r*(74) = −0.30, *p* < 0.05]. Aggression was inversely associated with plasma oxytocin levels in participants of the homicide group [*r*(32) = −0.61, *p* < 0.001], but was non-significant in participants of the control group [*r*(73) = −0.01, *p* = 0.94].

### Mediation Analysis

**Figure 2:**
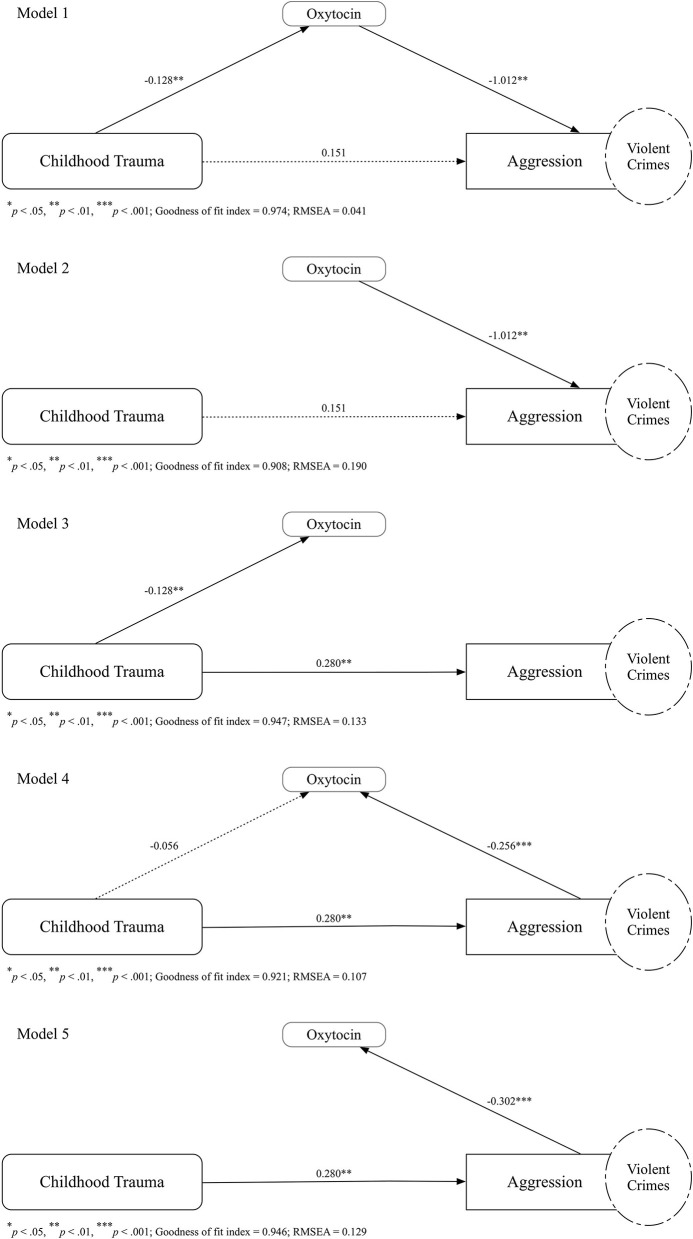
Path diagram illustrating the unstandardized regression coefficients for the relationship between childhood trauma and aggression, as mediated by levels of plasma oxytocin in persons convicted for homicide. Several theoretical hypotheses of the relationship between childhood trauma, aggression, and plasma oxytocin levels have been postulated: (Model 1) the relationship between childhood trauma and aggression was mediated by oxytocin, (Model 2) childhood trauma and oxytocin both contributed to aggression independently, (Model 3) childhood trauma was linked to both oxytocin and aggression independently, (Model 4) both childhood trauma and aggression contributed to changes in oxytocin and the childhood trauma also leading to aggression, and (Model 5) childhood trauma was linked to aggression while aggression subsequently promoting changes in oxytocin. The numbers above the arrows indicate the unstandardized regression coefficients of the path in the model. Fitness of the model was examined using a goodness of fit index (GFI) ≥0.90, and a root mean square error approximation (RMSEA) value of ≤0.06. The Model 1, as the best-fit model (GFI = 0.974; RMSEA = 0.041), shows that the effect of childhood trauma on aggression was mediated by plasma oxytocin levels among participants in the homicide group.

A mediation analysis was further performed for participants in the homicide group. The overall mediation analysis of plasma oxytocin levels is illustrated in Figure 2. After considering all criteria in this study, Model 1 was selected as the best-fit model (GFI = 0.974; RMSEA = 0.041). For participants in the homicide group, the regression coefficient between childhood trauma and plasma oxytocin levels was statistically significant (*p* < 0.01), as was the regression coefficient between plasma oxytocin levels and aggression (*p* < 0.01). The bootstrapped unstandardized indirect effect was non-significant (β = 0.13, *SE* = 0.06, 95% CIs [0.02, 0.25]). The effect of childhood trauma on aggression was fully mediated by plasma oxytocin levels among participants in the homicide group. All other covariates proposed in this study, including age (*p* = 0.76), height (*p* = 0.98), body weight (*p* = 0.27), and cigarette smoking (*p* = 0.50), did not interfere with regression coefficients in the meditational model.

## Discussion

The results indicate that traumatic experiences in childhood were higher in the persons convicted for homicide than among those in the control group without criminal convictions. In particular, persons convicted for homicide had a higher prevalence of physical abuse during childhood, than control group participants in this study. Despite the widespread prevalence of childhood trauma, less is known about its biological import. Humans are irrevocably shaped by their developmental environment through the biological imprinting of early experiences (47). The current study demonstrated a positive correlation between childhood trauma and aggression, with plasma oxytocin levels inversely correlated with both factors. Although a history of childhood trauma appears to contribute to the use of violence, few studies have explored the nature of this relationship and its association with hormonal changes. Reduced endogenous oxytocin levels in people with childhood trauma were also observed in previous meta-analyses, supporting the hypothesis that early adversity persistently alters oxytocin production and release in adulthood (48).

The role of oxytocin in the mediation model is notable. Several mediation models have been postulated and the main differences between the five models are the path directions between plasma oxytocin levels and aggression. In Model 1 and Model 2, plasma oxytocin levels are proposed to have contributed to the later aggression. In Model 4 and Model 5, aggression is proposed to promote the changes in plasma oxytocin. Model 1, as the best-fit model, shows that the effect of childhood trauma on aggression was mediated by plasma oxytocin levels among participants in the homicide group. The bidirectional interaction between aggression and oxytocin that is demonstrated in different mediation models may add to the ongoing debate questioning the unidirectional prosocial role of oxytocin (36). However, in this study, the models other than Model 1 are rather satisfied to examine their mediation path, yet, still too early to draw any conclusion for the other models. In this study, the mediation analysis demonstrated that those with higher childhood trauma scores had lower plasma oxytocin levels, which may have led to them having higher aggression levels. Oxytocin lowers the cortisol levels that increased in the hypothalamus–pituitary–adrenal axis for stress response. Dysregulation of oxytocin inhibition process caused by a failure to exhibit high oxytocin receptor levels in the amygdala of individuals who have experienced trauma allowing stress responses to escalate to unproportionally high levels (13). By reducing the amygdala activation with supplementation of exogenous oxytocin has been shown to reduce danger signaling, promote trust, increase sociability, decrease social fear, and possibly attenuate aggression (22).

In this study, the effect of childhood trauma on aggression was mediated by plasma oxytocin levels in persons convicted for homicide; however, the mediation effect could not be verified among participants in the control group. Differences between these two groups of participants may imply some hidden factors contributing to, or preventing, the occurrence of aggressive behaviors. In other words, the proposed hypothesis regarding the association between oxytocin level and the attachment of individuals with experiences of childhood trauma, did not fully explain the results found in this study. One possible explanation is the oxytocin's dose-dependent variability in aggression. Acute administration of oxytocin produces dose-dependent changes in reducing aggression in rats (49). The degree of oxytocin deprivation has to exceed the threshold to become violent is a probable factor that resulted in this disparity across participants. The concept of resilience may also explain the discrepancy between different sequela faced by individuals with experiences of childhood trauma. The timing of exposure to childhood trauma may result in different developmental sequelae (50). For example, earlier exposure to maltreatment was associated with a blunted amygdala response and failure to centrally upregulate oxytocin receptors, reducing sensitivity to adaptational fight-or-flight reactions that promote survival (51). Besides, several studies have focused on adaptive coping styles and personal attributes, such as ego strength, tenacity, self-efficacy, and cognitive flexibility, related to resilience, that appear to mitigate negative sequelae in response to childhood trauma (52, 53). In addition to coping styles and personal attributes, resilience may be predicted by “gene × environment” interactions with childhood trauma. Studies showed that only individuals surrounded by a positive family environment during childhood (54) and higher school connectedness (55) were found to have increased resilience in adulthood, likely as a result of a heightened sense of belonging (56). Although they have lower resilient functioning than those without childhood trauma, traumatized children nonetheless strive to be resilient. A strong moderating effect from having a positive social environment was identified in adults with a specific allele of the oxytocin receptor gene, *OXTR*, who had been exposed to early childhood trauma (9). Childhood trauma has been shown to consistently exert strong and adverse effects on the resilience of those who experience it; indicating that the path to aggression is mediated by oxytocin.

### Limitations and Future Research Directions

The strength of this study lies in providing relevant findings which further illustrate the role of oxytocin in linking childhood trauma to aggression. As with any study, the findings should be interpreted with consideration to recognized limitations. First, the validity and reliability of the retrospective self-reporting of childhood trauma carry their own caveats as, due to potential recall bias, possible under-reporting may lead to substantial measurement errors (57). Albeit the simple forgetting, non-awareness, and non-disclosure (19), retrospective reports of childhood trauma are still valuable for examining its association with adulthood adversities, such as psychiatric problems (58). In this study, participants were asked to provide in-depth clarification about their experiences of childhood trauma during interviews, for cross-validation, to minimize any aforementioned reporting biases. A prospective study design, with follow-up from baseline experiences of childhood trauma and oxytocin level changes, is necessary to confirm correlations more definitively among these factors.

Oxytocin is determined not only by childhood trauma, but also from other stressful events (59), inflammation (60), nicotine use (61), and the hypothalamus–pituitary–adrenal axis (62). Perhaps not only oxytocin but aggression is explained by the aforementioned factors besides childhood trauma. In interpreting the positive correlation between childhood trauma and plasma oxytocin levels, these unmeasured factors should be taken into account. Although it is impractical to sample the cerebrospinal fluid, using the peripheral plasma oxytocin level as a surrogate for central oxytocin function may raise questions about the accuracy of oxytocin measurement and its implications. The previous study had demonstrated a positive association does exist between central and peripheral oxytocin levels (63). Age-related patterns of oxytocin concentration are influenced by reproductive status, and plasma oxytocin levels are significantly higher in women than in men (64). Therefore, the enrollment of participants in this study was limited to men who had not undergone hormone therapy, to avoid the confounding effects of gender on the findings. This design restriction means that the results cannot be generalized to women.

Finally, given the study's cross-sectional design, this study could not isolate the effects of trauma exposure timing. This is an inherent design obstacle, as obtaining self-reports prospectively from young children is ethically inappropriate. As with most cross-sectional studies, a causal relationship cannot be determined unless assumptions are made. We tried to examine the possible links between childhood trauma, oxytocin, and aggression, by performing different structural equation models to establish the best fit. After careful modification and validation through statistical analysis, with the assumption that oxytocin concentration remained relatively stable across adulthood, this study sought to determine a path between childhood trauma and aggression and reveal the interaction of oxytocin within. Owing to limitations inherent in this cross-sectional design, it is recommended that in any future study, longitudinal relationships be examined to confirm causality more definitively over time.

## Conclusions

This study explored the pathway of oxytocin through which childhood trauma leads to later aggression in persons convicted for homicide. Fully elucidating the factors leading to homicide is difficult as crime involves a complex interplay between an individual and the society in which they grow up. Childhood trauma is correlated with aggression, whereas plasma oxytocin level is inversely correlated with childhood trauma. A theoretical framework has been postulated to explain this possible pathway, as experiencing childhood trauma decreases plasma oxytocin levels and subsequently contributes to higher aggression in persons convicted for homicide. Prevention and early identification of childhood trauma are crucial for reducing aggression in adulthood. A decrease in incidents of childhood trauma could decrease the risk of alterations in oxytocin gene expression and secretion. For those with severe childhood trauma experiences, efforts to activate secure attachment should be made promptly. We hope that the results of this study will lead to further theoretical consideration of how best to explain the interaction between childhood trauma and aggression, thus assisting to develop further preventive strategies.

## Data Availability Statement

The original contributions presented in the study are included in the article, further inquiries can be directed to the corresponding author.

## Ethics Statement

The studies involving human participants were reviewed and approved by Joint Institutional Review Board of Taipei Medical University. The participants provided their written informed consent to participate in this study.

## Author Contributions

KKG provided the ideation, collected and analyzed the data, and wrote the manuscript. M-LL had made a critical review of the manuscript. SJ assisted in interpreting the data and contributed to the writing of the manuscript. All authors have approved the final version of the manuscript.

## Conflict of Interest

The authors declare that the research was conducted in the absence of any commercial or financial relationships that could be construed as a potential conflict of interest.

## Publisher's Note

All claims expressed in this article are solely those of the authors and do not necessarily represent those of their affiliated organizations, or those of the publisher, the editors and the reviewers. Any product that may be evaluated in this article, or claim that may be made by its manufacturer, is not guaranteed or endorsed by the publisher.
